# Multi-Cause Calibration of Verbal Autopsy–Based Cause-Specific Mortality Estimates of Children and Neonates in Mozambique

**DOI:** 10.4269/ajtmh.22-0319

**Published:** 2023-04-10

**Authors:** Brian Gilbert, Jacob Fiksel, Emily Wilson, Henry Kalter, Almamy Kante, Aveika Akum, Dianna Blau, Quique Bassat, Ivalda Macicame, Eduardo Samo Gudo, Robert Black, Scott Zeger, Agbessi Amouzou, Abhirup Datta

**Affiliations:** 1 Department of Biostatistics, Johns Hopkins University, Baltimore, Maryland;; 2 Department of Biostatistics, Epidemiology and Informatics, University of Pennsylvania, Philadelphia, Pennsylvania;; 3 Department of International Health, Johns Hopkins University, Baltimore, Maryland;; 4 Center for Global Health, Centers for Disease Control and Prevention, Atlanta, Georgia;; 5 ISGlobal, Hospital Clínic - Universitat de Barcelona, Barcelona, Spain;; 6 Centro de Investigação em Saúde de Manhiça (CISM), Maputo, Mozambique;; 7 Catalan Institution for Research and Advanced Studies (ICREA), Barcelona, Spain;; 8 Pediatrics Department, Hospital Sant Joan de Déu, Universitat de Barcelona, Barcelona, Spain;; 9 Consorcio de Investigación Biomédica en Red de Epidemiología y Salud Pública (CIBERESP), Madrid, Spain;; 10 Instituto Nacional de Saúde (INS), Maputo, Mozambique

## Abstract

The Countrywide Mortality Surveillance for Action platform is collecting verbal autopsy (VA) records from a nationally representative sample in Mozambique. These records are used to estimate the national and subnational cause-specific mortality fractions (CSMFs) for children (1–59 months) and neonates (1–28 days). Cross-tabulation of VA-based cause-of-death (COD) determination against that from the minimally invasive tissue sampling (MITS) from the Child Health and Mortality Prevention project revealed important misclassification errors for all the VA algorithms, which if not accounted for will lead to bias in the estimates of CSMF from VA. A recently proposed Bayesian VA-calibration method is used that accounts for this misclassification bias and produces calibrated estimates of CSMF. Both the VA-COD and the MITS-COD can be multi-cause (i.e., suggest more than one probable COD for some of the records). To fully use this probabilistic COD data, we use the multi-cause VA calibration. Two different computer-coded VA algorithms are considered—InSilicoVA and EAVA—and the final CSMF estimates are obtained using an ensemble calibration that uses data from both the algorithms. The calibrated estimates consistently offer a better fit to the data and reveal important changes in the CSMF for both children and neonates in Mozambique after accounting for VA misclassification bias.

## INTRODUCTION

In Mozambique, the Countrywide Mortality Surveillance for Action (COMSA) platform provides continually updated statistics on mortality and cause of death (COD) for the country. The goal is to conduct COD analyses stratified by age and province level to inform the government of Mozambique and other stakeholders. This is important for the nation’s public health because Mozambique does not have a comprehensive civil registration and vital statistics system.

Countrywide Mortality Surveillance for Action has implemented a sample registration system (SRS) of births and deaths, but accurately determining the COD is challenging because many deaths occur outside hospitals. Countrywide Mortality Surveillance for Action has trained Community Surveillance Assistants (CSAs) deployed for interviews of families of the deceased individuals registered in the SRS. The CSAs conduct verbal autopsies (VAs), standardized series of questions that establish the health history and signs and symptoms of the fatal illness.^1^ These questionnaires can be examined by physicians to establish a likely COD,^2^ but such a protocol is costly and difficult to standardize. Instead, computer-coded VA (CCVA) algorithms like InSilicoVA,^3^ InterVA,^4^ expert algorithm (EAVA),^5^ Tariff or SmartVA,^6^ and naive Bayes classifier^7^ can be used to automatically infer COD.

Computer-coded VA data can be used directly to estimate cause-specific mortality fractions (CSMFs; i.e., the proportion of deaths attributable to a set of causes) at the population level. However, the outputs of the CCVA algorithms are merely statistical predictions and not definitive measurements. Any biases present in individual-level COD predictions will be propagated to the aggregated estimates (i.e., the population-level CSMF).

In this manuscript, we conduct analysis to obtain CSMFs for neonates aged 1–28 days and children aged 1–59 months in Mozambique using the COMSA VA data. There are a priori reasons to suspect the presence of large biases in the results from CCVA algorithms used for the COMSA VA data. For example, major CCVA algorithms have been trained on data from the Population Health Metrics Research Consortium (PHMRC). These data date to 2011 and were collected in countries other than Mozambique. Due to varying causes of disease and cultural differences in the communication underlying the VA methods, the relationship between reported symptoms and underlying causes is likely substantially different between the PHMRC data and the COMSA data. Accuracy of CCVA is known to highly depend on the training data.^8^ This means CCVA algorithms that might be accurate in the PHMRC cohort may be quite biased in the COMSA cohort.

It has been demonstrated that biased CSMF estimates from CCVA algorithms may be improved by incorporating auxiliary data sources with more comprehensive COD information for the purpose of calibration.^9^^,^^10^ Specifically, we use a comparatively small set of data from the Child Health and Mortality Prevention (CHAMPS) project collected in Bangladesh, Ethiopia, Kenya, Mali, Mozambique, Sierra Leone, and South Africa.^11^ For deaths assessed in this project, a panel of experts determines the COD/chain of events leading to death using medical history and records of the terminal illness and post-mortem multiple pathogen screening and biopsy pathology data using minimally invasive tissue sample (MITS) procedures.^12^ The CODs determined by the CHAMPS process will be referred to as “MITS-COD,” recognizing that use of MITS is an important addition to improve the validity of medical certification of COD. The COD determination informed by MITS has been shown to be highly concordant with COD from full diagnostic autopsies.^13^ The deaths studied by CHAMPS also have VA data, and it is possible to obtain the CCVA-predicted COD, henceforth referred to as “VA-COD.” This paired dataset of MITS-COD and VA-COD allows us to estimate the misclassification rates of the CCVA algorithms in this cohort. Combining this evidence with the raw COMSA CCVA results using a Bayesian algorithm as implemented in the calibratedVA R package,^14^ we are able to provide calibrated CSMF estimates that account for the misclassification of causes by the CCVA methods. In addition, we provide results from an ensemble calibration algorithm that combines the input of multiple CCVA algorithms.

The VA calibration procedure is simplest when the CCVA and MITS autopsy results identify a single COD. However, CCVA algorithms like InSilicoVA and InterVA may output probabilistic predictions, suggesting multiple plausible CODs with an assigned score. Ignoring this uncertainty in COD prediction reflected in the multi-cause VA-COD output wastes information. To understand this, when probabilistic predictions are converted to single-cause predictions, a plurality rule is used (i.e., the cause with the highest score is assigned to be the VA-COD for that case). Therefore, a COD with relatively low probability may be assigned as the definitive cause. Additionally, for MITS results, many individuals are considered to have multiple CODs, including one underlying (the one precipitating the chain of events ultimately leading to death) and an immediate (the closest one to the fatal event) cause. A single-cause analysis would only use the underlying cause, missing important information on the causal chain of illnesses leading to death.

Due to the multi-cause nature of both the VA-COD and MITS-COD data, we implement a multi-cause procedure for CCVA calibration as described by Fiksel et al.,^9^ which sensibly incorporates multiple causes in both the VA and the MITS results. This is accomplished through a novel generalization of the notion of misclassification rates for multi-cause data^15^ and by using a “generalized Bayes” estimation technique that replaces the full probability likelihood model with the solution of an estimating equation incorporating a loss function^16^^,^^17^ because the latter is easier to deal with for multi-cause data.

Two different CCVA algorithms, InSilicoVA and EAVA, are considered for the analysis, and the final CSMF estimates are obtained using an ensemble calibration that uses data from both the algorithms. We show that use of the multi-cause COD output improves the sensitivities of both the CCVA algorithms with respect to the MITS-COD. The calibrated CSMFs show significant differences from the uncalibrated ones and offer substantially improved fit to the data.

## MATERIALS AND METHODS

### Overview of single-cause VA-calibration.

To estimate the prevalence of various CODs in Mozambique, we have the results of VAs for 1,841 child deaths (aged 1–59 months) and 818 neonatal deaths. These autopsies are analyzed by two CCVA algorithms of inherently different nature – InSilicoVA,^3^ which assigns conditional probabilities of COD, and EAVA,^5^ an algorithm that follows logical rules related to reported signs and symptoms of the fatal illness to move through a hierarchical decision tree to assign a COD.

To introduce the calibration procedure, we will begin by assuming that one COD is identified by the algorithms. In the case of InSilicoVA, this is accomplished by selecting a cause by “plurality rule” (i.e., the COD with the highest predicted probability). EAVA, by default, offers only a single COD. Given these predictions from either of these algorithms, we can estimate the CSMF with the sample proportions.

However, these uncalibrated CSMF estimates will exhibit substantial bias due to systematic misclassification in the VA predictions. For each pair of causes *i,j* (and a specific algorithm, InSilicoVA, or EAVA), *M_ij_* denotes the rate that a subject with true COD *i* (as diagnosed by a more comprehensive diagnostic procedure like MITS) will be predicted as having COD *j* by the CCVA algorithm. If *i* = *j*, then *M_ij_* is the probability of a correct classification of an individual with condition *i*. Otherwise, *M_ij_* is the probability of misclassifying such an individual as having condition *j*. Stacking up the unknown *M_ij_*’s into a matrix, we have a parameter *M* that represents the misclassification rates of the CCVA algorithm. *M* will be close to the identity matrix (with ones on the diagonal and zeros elsewhere) for an accurate algorithm and far from the identity matrix for an inaccurate algorithm.

To overcome the bias in raw CSMF estimates, we estimate the misclassification rates *M* for each of the two CCVA algorithms with respect to the MITS-COD. The CHAMPS data used in this analysis contains MITS-COD for the deaths of 426 children and 614 neonates across all sites. For the same subjects, we have the results of the automated algorithms (EAVA and InSilicoVA) run on their VA and can obtain the VA-COD for the respective algorithms. These paired data are used to estimate the misclassification rates. We do not assume that the cause-specific COD proportions are the same for the COMSA and CHAMPS, but rather that the conditional misclassification rates of the VA algorithms with respect to MITS-COD are equivalent between the two.

We first illustrate how the calibration works using a simple example with three causes. Let (*p*_1_, *p*_2_, *p*_3_) denote the true CSMF for these causes in the population of interest and (*q*_1_, *q*_2_, *q*_3_) denote the uncalibrated CSMF estimated from a CCVA algorithm. One can obtain an estimate of the error rates of the algorithm from a paired dataset of VA- and MITS-COD. These rates are summarized in an error matrix *M* = (*M_ij_*), where *M*_11_ is the sensitivity of VA identifying the first cause, *M*_21_ is the error rate of VA-COD being cause 1 when the MITS-COD is cause 2, and the other entries are similarly defined. Then, following the law of total probability, we have

$$\begin{array}{l} q_{1}=P\left(\text{VA COD}=\text{cause }1\right)\\ =P\left(\text{MITS COD}=\text{cause }1\right)*P\left(\text{VA COD}=\text{cause }1|\text{MITS COD}=\text{cause }1\right)\\ \;+P\left(\text{MITS COD}=\text{cause }2\right)*P\left(\text{VA COD}=\text{cause }1|\text{MITS COD}=\text{cause }2\right)\\ \;+P\left(\text{MITS COD}=\text{cause }3\right)*P\left(\text{VA COD}=\text{cause }1|\text{MITS COD}=\text{cause }3\right)\\ =p_{1}*M_{11}+p_{2}*M_{21}+p_{3}*M_{31} \end{array}$$

We can develop similar equations for *q*_2_ and *q*_3_. To generalize this to the case with more than three causes, we denote by *p* the target parameter of interest (i.e., the population CSMF); *p* is a vector whose *i*^th^ component is the CSMF for the *i*^th^ cause. We let *q* denote the apparent CSMF as provided by the raw (uncalibrated) and biased CCVA algorithms. Following the law of total probability, the apparent CSMF for each cause *j* is a weighted estimate of the true CSMF for all causes, weighted by the proportion of times those causes are misclassified as cause *j* by CCVA. In mathematical terms, this yields the equation

q=M′p (1)

Note that *q* and *M* are both directly estimable from the available data: *q* is measured by the aggregated COMSA raw CSMF estimates from the CCVA algorithms, and *M* is measured by comparing the CCVA and CHAMPS results. If *q* and *M* were known with certainty, then *p* could be calculated by solving the system of linear equations *q* = *M*′*p*. Because *q* and *M* instead are associated with statistical estimates based on data, one can use a Bayesian procedure for the calibration as described by Datta et al.^10^ The approach uses multinomial likelihoods for the data and conducts Bayesian inference using Markov Chain Monte Carlo that ensures propagation of uncertainty.

### Multi-cause CCVA outputs.

As mentioned earlier, for each VA record, the InSilicoVA algorithm outputs predicted conditional COD probabilities. Although this prediction can be summarized into one class (the class with the highest predicted probability), such a procedure wastes information. For example, it considers deaths with a 60% probability of a COD to contain the same information as deaths with a 100% probability, even though the latter is clearly stronger evidence for that COD. Also, with more than two CODs, the largest predicted probability could even be well under 60%. In addition, a disease that shares a symptom profile with a more common COD might never be identified as the most likely COD in any individual case; the single-cause procedure will incorrectly indicate a prevalence of zero for such a disease. We aim instead to use the multi-cause output of complete set of InSilicoVA predicted probabilities for each individual case, without recourse to the plurality rule.

EAVA is a deterministic algorithm designed to yield single-class COD predictions. We used a novel modification of the EAVA algorithm to generate multi-cause predictions for cases where more than one COD is compatible with the VA responses. This is achieved by running the algorithm first normally to identify the most likely cause, then running the algorithm a second time with the most likely cause removed from the COD hierarchy. The cause selected by this second run of the algorithm is identified as the second most likely cause. We then create a multi-cause EAVA output assigning the most likely cause a probability of 75% and the second most likely cause a probability of 25%. All other causes are assigned a probability of 0%. Sensitivity analysis was conducted to study the impact of the choice of the weights.

### MITS underlying and immediate causes.

Death is a complex process involving a causal chain encompassing multiple causes. In fact, for each death assessed by the CHAMPS process that is enrolled with MITS, all CODs are captured, which includes both an underlying and immediate cause being identified. The single-cause analysis would only use the “underlying” cause because it is the first one to appear and leads to the chain of events causing death. However, in many deaths, the immediate cause, besides being the final cause in the causal chain, carries important information that may affect policy or clinical decisions. For example, if we only know that deaths had HIV (underlying cause) and ignored that the terminal events involved tuberculosis or pneumonia, we would not be well informed. Therefore, we aim to allow the use of up to two MITS-CODs (underlying and immediate) for each death (recognizing that there may be even more causes for some deaths). If MITS does not identify two distinct causes (i.e., if both the underlying and immediate causes are the same), the MITS-COD remains a single cause as before.

### Multi-cause misclassification matrix.

With these aims in mind, it is necessary to generalize the notion of CCVA misclassification rates described above to allow for multiple causes for both the CCVA outputs and the CHAMPS diagnosis. We first extend the definition of misclassification matrix for multi-cause CCVA outputs but with single-cause MITS output. Recall that for a single-cause analysis, each entry of the misclassification matrix *M_ij_* is the proportion of deaths that belong to class *i* (i.e., have MITS-COD *i*) that are identified as belonging to class *j* (i.e., have VA-COD *j*). For multi-cause CCVA output, the misclassification rate *M_ij_* is defined as the average score assigned to cause *j* by the CCVA algorithm among all deaths that would be attributed to MITS-COD *i*. Because for binary data averages are the same as proportions, the multi-cause definition of misclassification rates agrees with the previous definition of *M* if all the CCVA outputs were single cause.

The calibration framework also allows for multiple MITS causes (underlying and immediate causes). This extension follows from recognizing that for multi-cause MITS-COD, the VA-COD for a death is a mixture of the misclassification rates of CCVA for the two possible MITS-COD (underlying and immediate) for that death. To formally understand this, for a total of *C* causes considered, we can summarize the multi-cause MITS-COD for a case in a *C*-length vector *x*. The entries of *x* will be 1 (if a cause is identified as both the underlying and immediate causes of death), 0.5 (if the cause is identified as one of immediate or underlying MITS-COD but not both), or 0 (if the cause is not identified as either COD). Consider a case where MITS identifies cause 1 as the immediate cause and cause 2 as the underlying cause. This MITS diagnosis can be expressed as a *C*-length vector *x* = (0.5, 0.5, 0, …, 0). The multi-cause calibration interprets the MITS diagnosis as assigning 50 out of 100 individuals with such a MITS diagnosis to cause 1 and the remaining 50 individuals to cause 2. Hence, the proportion of times the CCVA will predict cause *j* for such a case will be the weighted average of the VA misclassification rates for causes 1 and 2 (i.e., 0.5 * *M*_1_*_j_* + 0.5 * *M*_2_*_j_* = *M*′*x*). Thus, estimating the misclassification matrix corresponds to a linear regression *y *= *M*′*x *of the multi-cause VA-COD *y* on the multi-cause MITS-COD *x*, and the misclassification matrix *M* can be interpreted as the multi-dimensional regression line. This notion of the misclassification matrix is formally defined by Datta^15^ and implemented in the codalm R package.^18^ Note that if the MITS identifies a single cause (i.e., one entry of *x* is 1), then this formulation agrees with the previous single-cause definition of *M*.

### Multi-cause calibration.

The generalization of the misclassification matrix allows us to extend the VA calibration to allow multi-cause VA-COD and MITS-COD. The CHAMPS data of paired MITS and VA records are used to estimate the misclassification matrix using the regression method described in “Multi-cause misclassification matrix.” The COMSA VA data allows estimating the raw (uncalibrated) CSMF *q*. Letting *p* denote the calibrated CSMF, the equation *q *=* M*′*p* presented in “Overview of single-cause VA-calibration” remains valid for multi-cause data. Hence, subsequent to estimation of *M* and *q*, one can solve for *p* using the equation above. However, unlike the single-cause data, which is categorical (discrete) and amenable to multinomial modeling, the multi-cause data (also termed compositional or fractional data) cannot be modeled using a multinomial distribution. This is because both the CCVA and MITS COD outcomes are no longer discrete variables. Multi-cause COD are now compositional variables (i.e., vectors of probability scores, with each probability representing the chance that death occurred due to the corresponding cause, summing up to 1).

Rather than maximizing a likelihood function, we minimize a loss function to connect parameters to the multi-cause data. The Kullback-Leibler divergence, or relative entropy loss, is a popular measure of dissimilarity connecting compositional or multi-cause data to parameters. Previous research provides a guide for Bayesian-style inference using loss function rather than a full likelihood.^16^^,^^17^ With a given loss function and prior, we have the generalized Bayes posterior for multi-cause VA calibration:

Generalized posterior∝exp[−loss(parameters|data)]∗prior

Hence, instead of a likelihood formulation, the multi-cause calibration is conducted in a generalized Bayes rule by using the relative entropy (Kullback-Leibler) loss functions for multi-cause data (see Fiksel et al.^9^ for details). This loss agrees with the multinomial likelihood for single-cause data, and thus the multi-cause VA calibration is a generalization of the single-cause one. With this set-up, computations to find the optimal estimates of unknown parameters can proceed in a manner analogous to the single-cause paradigm. To arrive at an ensemble calibrated estimate, the loss function is taken as the sum of the loss functions for EAVA and InSilicoVA. The purpose of combining the loss functions for both algorithms is to yield estimates that are consistent with the results of both algorithms. Note that taking the sum of the loss functions for the ensemble calibration is equivalent to placing equal importance to each VA algorithm. If there is a priori knowledge that one algorithm is more accurate than the other, one can also consider a weighted sum of the loss functions, placing higher weights to the more accurate algorithm. However, it is hard to quantitatively assess superiority of an algorithm a priori and assign a number to this. Also, although the prior weights are equal for the ensemble calibration, the final estimated CSMF will often tend to align with the estimate from calibrating the most accurate CCVA algorithm.^9^^,^^10^

Previously, extensive validation studies were presented using the benchmarking PHMRC dataset for the assessment of the multi-cause VA calibration method. These validation studies compared the calibrated and uncalibrated CCVA algorithms using the cause-specific mortality fraction accuracy (CSMFA) metric.^19^ However, this metric requires knowledge of the true CSMF for the dataset. This is available for the PHMRC dataset, which also contains a more comprehensive COD information for each record in addition to the VA data. For the COMSA analysis, the true CSMF is unknown and is the quantity of interest that needs to be estimated. Hence, CSMFA cannot be calculated.

To evaluate statistical models without knowledge of the true parameter values, a common strategy is to compare the out-of-sample prediction performance of the candidate models on hold-out data. This strategy cannot be adopted for VA calibration because the algorithm does not work at the individual level but only at the population level. In other words, the calibration does not calibrate the COD for the individual deaths or produce individual-level calibrated COD predictions; it only calibrates the estimate of the population-level CSMF. To compare uncalibrated models with their calibrated counterparts, we use the Widely Applicable Information Criterion (WAIC),^20^ a goodness-of-fit measure that uses in-sample model fit to estimate the model’s ability to predict future observations.^21^ WAIC provides an estimate of out-of-sample (prediction) error but using only in-sample (training) data.^22^ WAIC also harmonizes well with Bayesian inference, making use of the entire posterior distribution available from the Markov Chain Monte Carlo runs. Calculating the WAIC for the multi-cause calibrated models is not substantially different from calculating the WAIC for the single-cause calibrated models, except that the log-likelihood function is replaced with the negative log of the loss function.

### Data adjustments and exclusion.

Given the raw output of the MITS and CCVA algorithms, we have instituted a few steps of pre-processing to ensure the accuracy and validity of our procedure. Because conditions for neonates in South Africa hospitals differ from those in the other countries of interest, we exclude these 274 South Africa neonates from our analysis. The EAVA algorithm is inconclusive for a significant proportion of individuals, failing to identify any particular COD. These deaths would have to be by necessity excluded from a single-cause analysis, but the multi-cause calibration accommodates imputed values for this data with best available estimates. For the COMSA data, inconclusive EAVA deaths are assigned the average EAVA scores of all other (conclusive) EAVA scores. For a death with inconclusive EAVA diagnosis in the CHAMPS data, we impute the EAVA COD as the row of the EAVA misclassification matrix corresponding to the subject’s MITS underlying COD, where the misclassification matrix is calculated relative to the single-cause MITS. These choices are made to represent our best estimate of how the EAVA algorithm would classify such subjects. Because the imputed values would be multi-cause in nature, they cannot be included in the single-cause analysis. Thus, deaths with inconclusive EAVA diagnoses, which had to be removed from a single-cause analysis, are assigned multi-cause imputed scores, which are then used in the multi-cause calibration.

With *C* CODs, learning the misclassification matrix requires estimating a total of C*(*C* − 1) parameters. To minimize the dimensionality of the problem while retaining its important aspects, we group some CODs into more general labels based on evidence on the predominant CODs among children and neonates.^23^^,^^24^ For the children, we use the following seven classifications: malaria, pneumonia, diarrhea, severe malnutrition, HIV, other infections (which includes meningitis, typhoid fever, hepatitis, etc.), and other. For the neonates, we use the following five classifications: congenital malformation, infection (neonatal tetanus, meningitis and encephalitis, diarrhea, pneumonia, and sepsis), intrapartum-related events (IPREs), other, and prematurity.

## RESULTS

### Raw data.

The timeframe for the COMSA VA dataset used for all the analysis presented here is May 2018 to May 2021. Table 1 summarizes the CSMF results using the InSilicoVA and EAVA algorithms for children who died in the COMSA surveillance area. The estimated prevalences and the rankings of the causes are different for the two algorithms; the largest difference is that InSilicoVA recognizes many more malaria deaths than EAVA.

**Table 1:** Raw multi-cause counts and percentages in children (1–59 months) as predicted by InSilicoVA and EAVA using 1,841 COMSA children (1–59 months) VA records

	Malaria	Pneumonia	Diarrhea	Severe malnutrition	HIV	Other	Other infections
InSilicoVA, *n* (%)	356.8 (19.4)	275.7 (15.0)	445.2 (24.2)	75.6 (4.1)	58.3 (3.2)	199.6 (10.8)	429.9 (23.3)
EAVA,* n* (%)	144.2 (7.8)	437.1 (23.7)	344.4 (18.7)	148.3 (8.1)	116.1 (6.3)	106.6 (5.8)	544.2 (29.6)

COSMA = Countrywide Mortality Surveillance for Action; VA = verbal autopsy.

Table 2 summarizes the results for 818 neonatal deaths in COMSA for the same algorithms. The prevalence of infection is quite high for both algorithms, accounting for a majority of deaths by EAVA. IPRE and prematurity also account for large percentages of deaths. Congenital malformation is rare in the judgments of both algorithms.

**Table 2:** Raw multi-cause counts and percentages in neonates as predicted by InSilicoVA and EAVA using 818 COMSA neonate VA records

	Congenital malformation	Infection	IPRE	Other	Prematurity
InSilicoVA, *n* (%)	1.6 (0.2)	363.8 (44.5)	221.6 (27.1)	33.5 (4.1)	197.5 (24.1)
EAVA, *n* (%)	29.1 (3.6)	479.5 (58.6)	144.3 (17.6)	32.7 (4.0)	132.3 (16.2)

COSMA = Countrywide Mortality Surveillance for Action; IPRE = intrapartum-related event; VA = verbal autopsy.

Table 3 is a contingency table of the relationship between underlying (labeled horizontally) and immediate (labeled vertically) CODs of children as identified by CHAMPS. Because there are many entries off the diagonals, we can see that many deaths have multiple MITS causes.

**Table 3:** Distribution of CHAMPS MITS underlying and immediate causes of death of 426 children (1–59 months)

	Immediate cause								
		Malaria	Pneumonia	Diarrhea	Severe malnutrition	HIV	Other	Other infections	Total
Underlying cause	Malaria	34	8	0	0	0	5	4	51
	Pneumonia	0	27	1	0	0	4	14	46
	Diarrhea	0	9	15	0	0	0	4	28
	Severe malnutrition	9	18	6	0	0	2	35	70
	HIV	6	17	4	0	1	1	18	47
	Other	3	33	0	1	0	39	54	130
	Other infections	0	9	0	0	0	12	33	54
	Total	52	121	26	1	1	63	162	426

CHAMPS = Child Health and Mortality Prevention; MITS = minimally invasive tissue sampling.

We can see that pneumonia and other infections are relatively common as immediate causes, whereas severe malnutrition and other are more common as underlying rather than immediate causes. The combination of other as an underlying cause and other infections as an immediate cause is particularly frequent.

Table 4 is the contingency table of MITS underlying and immediate causes for the neonate cohort. We can see that for most of the causes the number of deaths where they were the underlying cause (row totals) are similar to the number of deaths where they were the immediate cause (column totals). The combination of infection as an underlying cause and prematurity as an immediate cause is relatively frequent.

**Table 4:** Distribution of CHAMPS MITS underlying and immediate causes of death of 340 neonates (excluding data from South Africa)

	Immediate cause						
		Congenital malformation	Infection	IPRE	Other	Prematurity	Total
Underlying cause	Congenital malformation	10	6	5	2	3	26
	Infection	1	61	4	1	26	93
	IPRE	4	13	118	2	10	147
	Other	0	2	2	7	0	11
	Prematurity	0	6	10	3	44	63
	Total	15	88	139	15	83	340

CHAMPS = Child Health and Mortality Prevention; IPRE = intrapartum-related event; MITS = minimally invasive tissue sampling.

**Figure 1.**
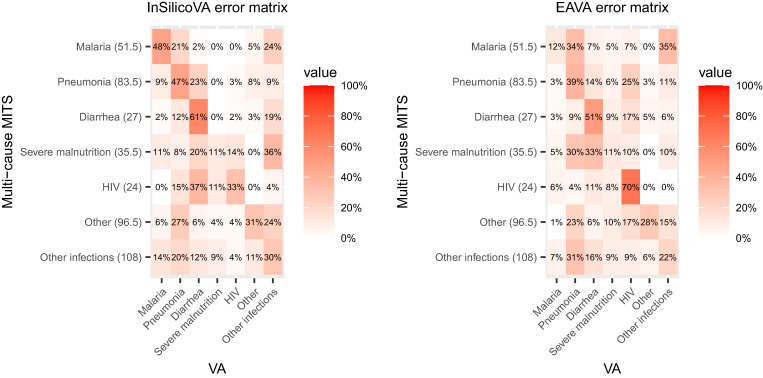
Pseudo-maximum likelihood estimates of misclassification rates matrices for multi-cause InSilicoVA and EAVA for children (1–59 months) based on the multi-cause Child Health and Mortality Prevention minimally invasive tissue sampling (MITS) data. The row totals indicate the sum of aggregate counts for MITS underlying and immediate causes of death.

Figure 1 displays the estimate of the multi-cause misclassification matrix *M* comparing the MITS-COD (row headers) and each VA-COD (column headers). Conditional on the MITS-COD classifications, each row gives the frequency of VA-COD of individuals with that MITS-COD for both InSilicoVA and EAVA. Because the diagonal entries are far from 100%, it is apparent that there is a high frequency of misclassification. Only a few CODs are correctly identified in more than half of the deaths (diarrhea by both algorithms and HIV by EAVA). Malaria is identified relatively often by InSilicoVA but not by EAVA. Severe malnutrition is misclassified very often by both algorithms.

**Figure 2.**
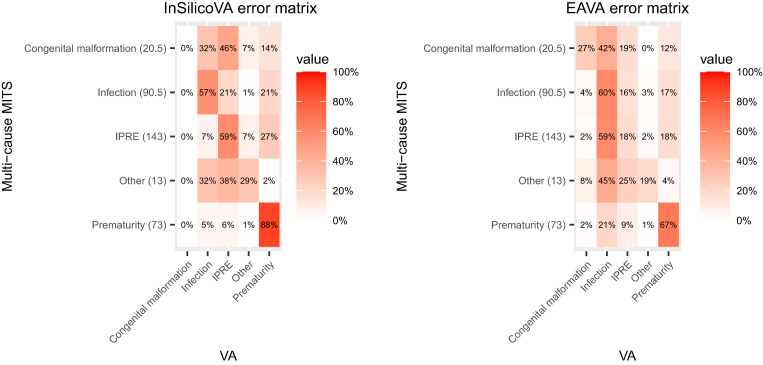
Pseudo-maximum likelihood estimates of misclassification rates matrices for multi-cause InSilicoVA and EAVA for neonates based on the multi-cause Child Health and Mortality Prevention minimally invasive tissue sampling (MITS) data. The row totals indicate the sum of aggregate counts for MITS underlying and immediate causes of death. IPRE = intrapartum-related event.

Figure 2 displays the same information for the neonatal deaths. Infection and prematurity are correctly identified in more than half the deaths by both algorithms, and IPRE is correctly identified by InSilicoVA for a majority of deaths. However, EAVA mislabels IPRE as infection in 59% of deaths. Note that congenital malformation, up to rounding error, is never identified as a COD by the InSilicoVA algorithm.

**Figure 3.**
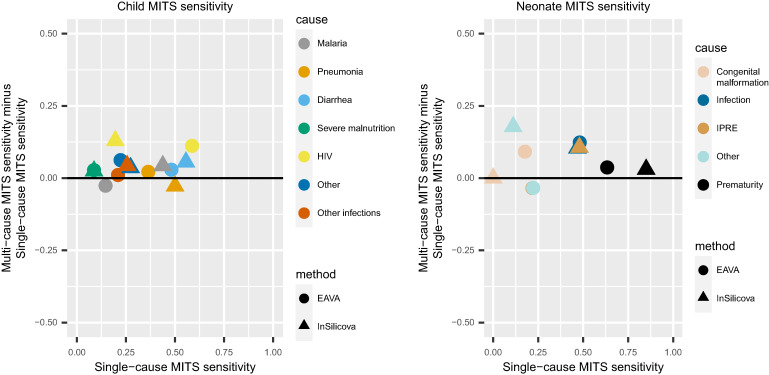
Comparison of single-cause and multi-cause estimates of true positive rates (sensitivities) of InSilicoVA and EAVA for children (1–59 months) (left) and neonates (right). The x-axis plots the sensitivities from the single-cause calibration; the y-axis plots the increase (or decrease) in sensitivity when switching to the multi-cause calibration. MITS = minimally invasive tissue sampling.

Figure 3 compares graphically the diagonal entries (i.e., the sensitivity values) of the single-cause and multi-cause misclassification matrices. We see that for both EAVA and InSilicoVA and for both children and neonates, the data points generally lie above the 45 degree line. We conclude that the multi-cause analysis has somewhat higher sensitivity. That is, according to the multi-cause weighting and misclassification calculations we have used, the MITS-COD and VA-COD are in better agreement for multi-cause data than the single-cause data. Causes where the multi-cause analysis leads to around 5% or more increased sensitivity for CCVA for children are HIV (both algorithms), diarrhea (InSilicoVA), and other (EAVA). For neonates, the multi-cause analysis leads to increased sensitivity for infection (both algorithms), IPRE (InSilicoVA), other (InSilicoVA), and congenital malformation (EAVA). For some of the other cause–algorithm combinations, this difference is not dramatic, and a few conditions (malaria for children; IPRE and other for neonates) are slightly better identified in the single-cause data for EAVA.

### Calibration.

**Figure 4.**
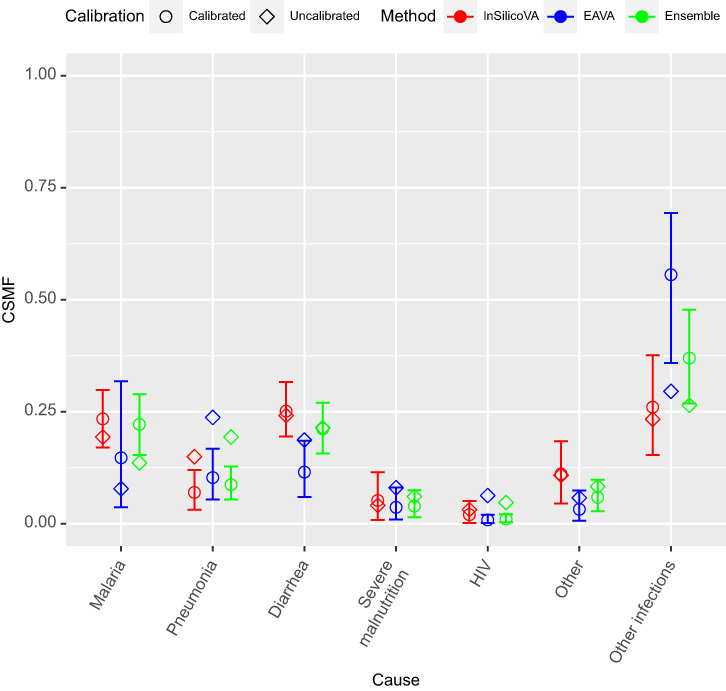
Multi-cause calibrated and uncalibrated cause-specific mortality fraction (CSMF) estimates for children (1–59 months) for the three verbal autopsy (VA) methods. The uncalibrated estimates of the CSMF are represented in diamonds, and the calibrated CSMF point estimates (the posterior means) are represented by circles. The vertical bars give the 95% credible intervals for the calibrated CSMF estimates. The three colors represents the three different VA algorithms: InSilicoVA (red), EAVA (blue), and ensemble (green).

We present the calibration results of the child deaths first. Supplemental Table 1 gives uncalibrated and calibrated CSMF estimates for InSilicoVA, EAVA, and the ensemble algorithm, with 95% credible intervals for the calibrated models. Figure 4 displays this information graphically. The pre- and post-calibration CSMF values are strongly related, yet there are some important differences. The calibration causes the estimated pneumonia CSMF to decrease for both algorithms; this can be understood from Figure 1 because pneumonia is identified with relatively high sensitivity but is often incorrectly identified in deaths that are attributable to other causes, particularly malaria (which is itself a common COD). Therefore, the calibrated model recognizes that many deaths that are identified as due to pneumonia by the CCVA should be properly allocated to other causes. Conversely, for the EAVA and ensemble algorithms, the estimated CSMF of other infection increases through calibration because sensitivity for this cause is low; the COD is often misclassified as pneumonia and diarrhea. Thus, the calibration recognizes that many deaths identified as due to pneumonia or diarrhea should properly be labeled as other infection. We can see that for EAVA the jump in the other infection CSMF after calibration is rather dramatic, but the CI is quite wide. This indicates high uncertainty in the posterior distribution, likely resulting from near-singularity of the misclassification matrix; that is, uncertainty in the estimation of the misclassification matrix causes even larger uncertainty in the estimation of the CSMF.

As reported in Supplemental Table 1, the calibrated CSMF estimates of the EAVA and InSilicoVA algorithms are similar, although InSilicoVA identifies more deaths as diarrhea and malaria, whereas EAVA identifies more deaths as other infection. As expected, the results of the ensemble algorithm that uses data from both InSilicoVA and EAVA are generally between the results of the two algorithms. However, the calibrated CSMF from the ensemble algorithm aligns much more closely with the calibrated InSilicoVA CSMF (Figure 4). In the plot of the error matrices for children in Figure 2, we see that the sensitivities for InSilicoVA are higher than those of EAVA for every cause except HIV. Therefore, the ensemble calibration agrees with the more accurate algorithm here. The ensemble model identifies other infection as the most common COD.

**Figure 5.**
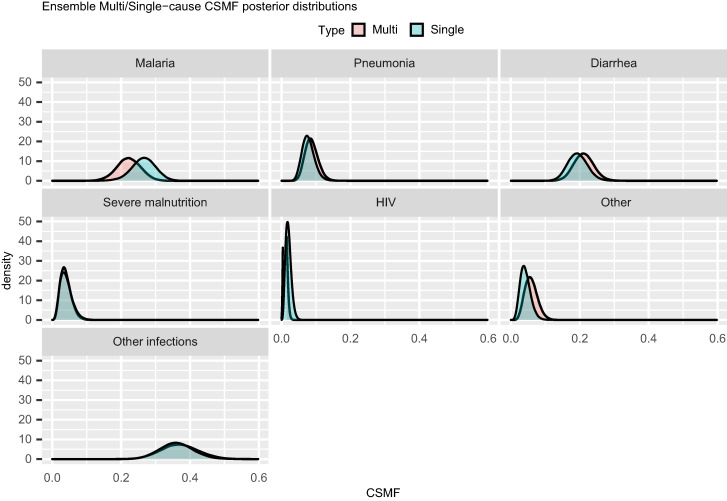
Comparison of the posterior densities of single- and multi-cause ensemble calibrated cause-specific mortality fraction (CSMF) for children (1–59 months).

**Figure 6.**
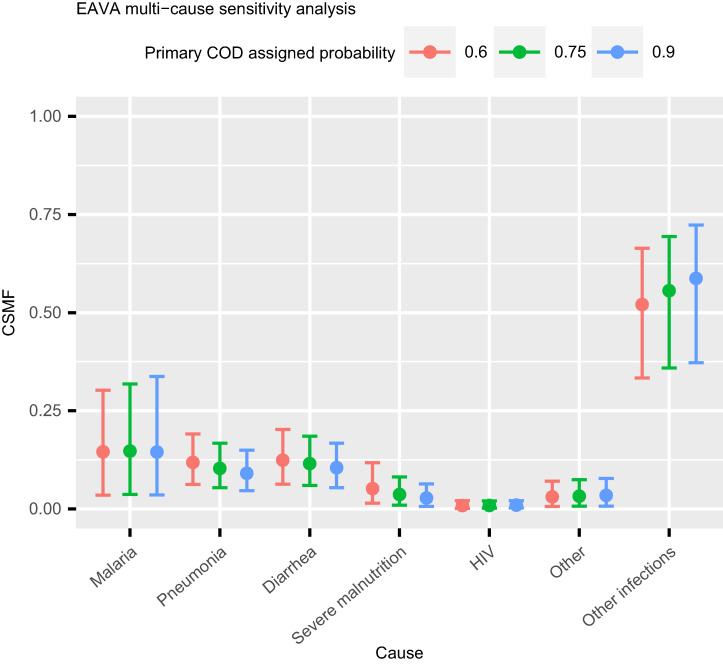
Sensitivity of child EAVA cause-specific mortality fraction (CSMF) point estimates and 95% CIs to adjustment of the assigned probability (weight) to the primary cause of death (COD) as identified by EAVA. (Main results use 0.75.)

Supplemental Figure 1 displays WAIC values for the calibrated and uncalibrated models for each algorithm. In each case, the calibrated model has a lower value of WAIC, indicating that the calibrated models are better fit to the data than the uncalibrated models. Figure 5 compares the posterior distribution of the ensemble CSMFs for the single-cause and multi-cause analysis. The analyses are largely in agreement, with some important changes. Malaria is identified as less prevalent in the multi-cause analysis, whereas diarrhea and other are identified as slightly more prevalent. Figure 6 indicates that results are not sensitive to the choice of probability weights assigned to the primary and secondary COD as identified by EAVA.

**Figure 7.**
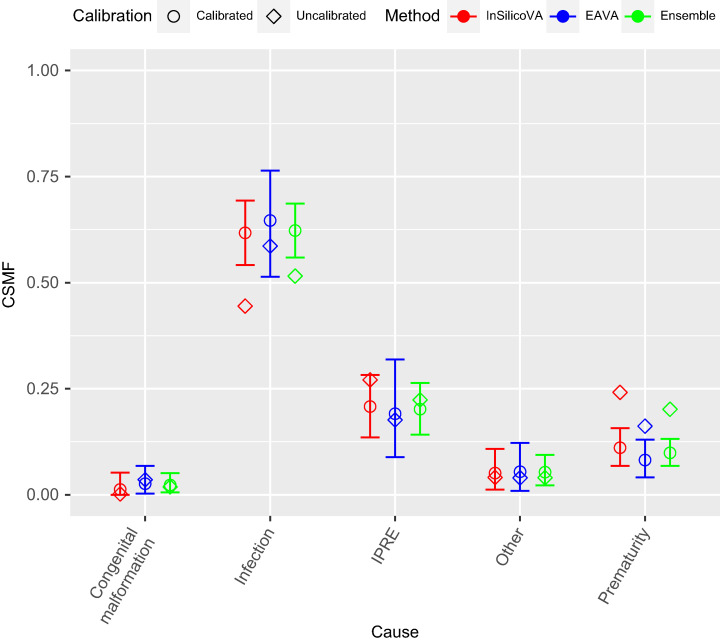
Multi-cause calibrated and uncalibrated cause-specific mortality fraction (CSMF) estimates for neonates for the three verbal autopsy (VA) methods. The uncalibrated estimates of the CSMF are represented by diamonds, and the calibrated CSMF point estimates (the posterior means) are represented by circles. The vertical bars give the 95% CIs for the calibrated CSMFs. The three colors represents the three different VA algorithms: InSilicoVA (red), EAVA (blue), and ensemble (green).

We discuss the findings for the neonatal deaths next. We can see from Figure 7 that the InSilicoVA CSMF for infection is increased through calibration. This is somewhat surprising because the sensitivity for the identification of infection is relatively high. However, true infection deaths are frequently mislabeled as causes that are common (prematurity and IPRE), whereas only uncommon causes (congenital malformation and other) are frequently labeled as infection, suggesting overall underreporting. This results in the increase in CSMF for infection after calibration. This increase in the CSMF for infection after calibration is less pronounced in EAVA because the increase is partly offset by adjusting for misclassification of a large percentage of MITS IPRE deaths as infection by EAVA. Calibration decreases the estimated CSMF of prematurity for both algorithms. This can be understood by noting that the sensitivity for prematurity is very high, and common causes (infection and IPRE) are frequently mislabeled as prematurity.

As given in Figure 7 and Supplemental Table 2, calibrated CSMF estimates of the EAVA and InSilicoVA algorithms are very similar, and the results of the ensemble algorithm are generally close to the average of the results of the two algorithms. All models identify infection as the most common cause of death. Supplemental Figure 2 indicates, as with the child data, that model fit is improved by calibration for all algorithms, as measured by WAIC. Supplemental Figure 3 demonstrates that the posterior distributions of CSMF are nearly identical for the multi- and single-cause analyses for neonates. Supplemental Figure 4 indicates that results are not sensitive to the choice of probability assigned to the primary COD as identified by EAVA.

## DISCUSSION

In this paper, we have described the application of a multi-cause VA calibration method to improve quantification of CSMF from CCVA data for child and neonatal deaths identified in COMSA. By cross-tabulating the results of CCVA algorithms with respect to MITS-COD, we can learn the misclassification patterns of the CCVA algorithms and thus correct the CSMF estimates. This paper has focused on the use of multiple causes in both the VA-COD and MITS-COD (up to two). We see this as preferable to the single-cause analysis because it more accurately incorporates the data sources because both VA-COD and the MITS-COD may identify multiple causes of death.

With respect to the COMSA data, we find that calibrated models are consistently better fits for the combined CHAMPS-COMSA data compared with their uncalibrated counterparts, as measured by WAIC. We find that, among other changes, calibration increases the estimated CSMF of malaria and other infections and decreases the estimated CSMF of pneumonia in children. For neonatal deaths, calibration increases the CSMF of infection and decreases the estimated CSMF of prematurity. We have attempted to intuitively explain these changes based on the misclassification matrices, but we note that the calibration involves solving a large system of equations and the point estimate of the misclassification matrix may not match the Bayesian estimates of the calibration procedure; thus, it may be difficult to understand the calibration results fully by intuition alone, and it is important to understand the basic principle of calibration, which is to adjust for imperfect sensitivities of the CCVA algorithms.

In the final results, infection is the most common cause of death in neonates, and other infections is the most common cause of death in children. Some historical data on CSMF for children and neonates in Mozambique are available from estimates published in the report of the INCAM VA survey in 2007 and those published in Perin et al.,^24^ which is informed by data from both the INCAM survey and COMSA. We see that for child deaths at 1–59 months, the multi-cause ensemble calibration estimated a higher CSMF for diarrhea (21%, with credible interval of 16–27%) compared with INCAM (6%) and Perin et al.^24^ (11%). This is due to InSilicoVA identifying a high proportion of diarrhea cases for COMSA data (Table 1). The CSMF for malaria was generally similar (> 20%) in all three estimates, as was the CSMF for pneumonia (∼10%). For neonates, both previous reports estimated a significantly higher CSMF of prematurity (INCAM: 35%, Perin et al.^24^: 48%) compared with the calibrated CSMF (10%, with credible intervals of 7–13%). We see from the error matrices in Figure 2 that both CCVA algorithms produced a large number of false positives for prematurity. The calibration adjusts for these overcounting of prematurity deaths, thereby reducing the CSMF for prematurity. This, in turn, increases the CSMF for infection for the calibration (62%, with credible interval of 56–69%), which is higher than the INCAM results (27%). The CSMF for IPRE is similar (∼20%) for all three sets of estimates.

There are several differences between our study and the INCAM study that make these estimates not directly comparable. They correspond to different time periods, are based on datasets that may not be comparable in terms of representativeness, and have used different methods for cause-of-death diagnosis. Therefore, the observed differences in estimates are possibly due to all of the aforementioned differences in the two settings. Neither the INCAM nor the Perin et al.^24^ estimates had considered VA misclassification, and adjusting for it presumably would have changed those results in the same way our raw results change after calibration. The calibrated CSMF from our study emphasizes need to account for this misclassification and to reevaluate the priorities for the health services based on the changes after calibration. However, before undertaking such actions, which of course would have resource implications, it would be important to seek confirmatory information of this bias of VA perhaps from health facility monitoring of deaths or even from demand for health care at facility level (and community, if there are services that could be monitored) for illnesses due to the same causes. The frequent misclassification of some causes by VA also has implications for care and the need to consider the causal chain (e.g., the possibility that the death in premature babies may be due to infection as highlighted in the large proportion of infection cases being misclassified as prematurity by VA) (Figure 2, left panel).

The current estimates use all the available COMSA data collected across the 11 provinces to produce a pooled calibrated CSMF estimate for Mozambique. One can also conduct the calibration on subsets of the data corresponding to specific provinces to produce subnational estimates. Such an exercise would be useful only if there is adequate sample size per province to yield estimates that are not too imprecise. Similarly, one can also stratify the analysis by time to produce yearly CSMF estimates and study trends over time. This would require the yearly subsets of the data to have uniform and representative geographical coverage across the country. We also expect the CSMF estimates to evolve with more COMSA and CHAMPS data collection and with the refinement of the CCVA algorithms.

The multi-cause calibration approach has certain limitations. The single-cause calibration is simpler to implement, but the assignment of multi-cause COD can be difficult using any method. Current implementation of the multi-cause calibration uses a simplified representation of the multi-cause MITS diagnosis by only using up to two MITS-COD (underlying and immediate causes). Future work will expand the multi-cause calibration framework to incorporate information more comprehensively about the entire set of (possibly more than two) causes present in the causal chain leading to the death. Also, both the single- and multi-cause calibration procedures rely on two main assumptions: 1) that the causes of death identified by MITS in the CHAMPS cohort are accurate and 2) that the CCVA misclassification rates in the COMSA population match the misclassification rates in the CHAMPS data. Future work needs to scrutinize these assumptions. For example, to understand representativeness of the MITS-VA error matrix estimated from the pooled CHAMPS data, it would be important compare error matrices from individual countries with MITS-VA pairs once enough MITS are conducted locally. If the MITS-VA error matrices reveal substantial heterogeneity across countries, it would highlight the need for a local dataset of MITS-VA pairs to estimate the VA misclassification rates.

The multi-cause analysis has important advantages over the single-cause analysis. Death is a complex process, and assigning any one cause, although it is simpler procedurally, can be problematic especially when using a probabilistic CCVA algorithm like InSilicoVA, which provides rich multi-cause output. Furthermore, multi-cause calibration allows use of all data, including the deaths where one of the CCVA algorithms is inconclusive. For such inconclusive deaths, the calibration requires imputation. The imputed value is a multi-cause COD estimate that cannot be used in a single-cause analysis. Thus, a single-cause analysis can lead to loss of a significant amount of data (in our case, single-cause analysis leads to loss of ∼15–20% of the COMSA data due to inconclusive EAVA diagnosis). We also see that the multi-cause analysis leads to better agreement between the VA-COD and MITS-COD (Figure 3). This is because the single-cause calibration, using only MITS underlying COD, would regard the deaths where VA-COD agrees with the MITS immediate COD but not with the MITS underlying COD as complete misclassifications. This would lead to higher estimates of misclassification. The multi-cause calibration considers such deaths as only partial misclassification and thus better captures the degree of false classifications in the CCVA algorithms.

For the COMSA-Mozambique data analysis, the final calibrated mortality fractions from the multi-cause analysis does not differ too much from the single-cause analysis for most causes. However, the advantages of the multi-cause analysis can manifest more in VA datasets from other populations. For example, if for a large fraction of deaths in the population the VA-COD is inconclusive between two causes but always assigns one cause a higher score than the other, then in a single-cause analysis the CSMF for the cause with lower score will always be zero, although it is likely that many of the deaths occurred due to this cause. Also, besides calibrating the VA-based CSMF, another future utility of the misclassification matrices is to help understand why the VA misclassifies such a large proportion of cases. For such a task, the multi-cause misclassification matrices help reduce cases with false misclassifications occurring due to either the single-cause VA or the single-cause MITS leaving out the matching cause. Hence, one can focus on studying cases with true mismatch between VA and MITS and try to improve the VA algorithms. Thus, it is advisable to use the multi-cause analysis rather than the single-cause one for studying and calibrating VA data.

## Financial Disclosure

Financial support: The COMSA Mozambique project is funded by the Bill & Melinda Gates Foundation (Grant no. OPP1163221).

## Supplemental Materials


Supplemental materials
