# Supplementary material for: Multi-Cause Calibration of Verbal Autopsy–Based Cause-Specific Mortality Estimates of Children and Neonates in Mozambique
Source: Am J Trop Med Hyg. 2023 Apr 10;108(5 Suppl):78–89. doi: 10.4269/ajtmh.22-0319 (PMC10160855; doi:10.4269/ajtmh.22-0319)
Supplement: Supplementary file 1 [file tpmd220319.SD1.pdf]

Supplement to “Multi-cause calibration of verbal autopsy-based cause-specific mortality estimates of children and neonates in Mozambique"

Table S1: Multi-cause raw (uncalibrated) and calibrated CSMF estimates (along with 95% confidence intervals) for U5 children from the 3 VA methods.

		Malaria	Pneumonia	Diarrhea	Severe malnutrition	HIV	Other	Other infections
InSilicoVA	Uncalibrated	19%	15%	24%	4%	3%	11%	23%
	Calibrated	23% (17% - 30%)	7% (3% - 12%)	25% (19% - 32%)	5% (1% - 11%)	2% (0% - 5%)	11% (5% - 18%)	26% (15% - 38%)
EAVA	Uncalibrated	8%	24%	19%	8%	6%	6%	30%
	Calibrated	15% (4% - 32%)	10% (5% - 17%)	12% (6% - 19%)	4% (1% - 8%)	1% (0% - 2%)	3% (1% - 7%)	56% (36% - 69%)
Ensemble	Uncalibrated	14%	19%	21%	6%	5%	8%	26%
	Calibrated	22% (15% - 29%)	9% (5% - 13%)	21% (16% - 27%)	4% (1% - 7%)	1% (0% - 2%)	6% (3% - 10%)	37% (27% - 48%)

Table S2: Multi-cause raw (uncalibrated) and calibrated CSMF estimates (along with 95% confidence intervals) for neonates from the 3 VA methods

		Congenital malformation	Infection	IPRE	Other	Prematurity

InSilicoVA	Uncalibrated	0%	44%	27%	4%	24%
	Calibrated	1% (0% - 5%)	62% (54% - 69%)	21% (14% - 28%)	5% (1% - 11%)	11% (7% - 16%)
EAVA	Uncalibrated	4%	59%	18%	4%	16%
	Calibrated	3% (0% - 7%)	65% (51% - 76%)	19% (9% - 32%)	5% (1% - 12%)	8% (4% - 13%)
Ensemble	Uncalibrated	2%	52%	22%	4%	20%
	Calibrated	2% (1% - 5%)	62% (56% - 69%)	20% (14% - 26%)	5% (2% - 9%)	10% (7% - 13%)

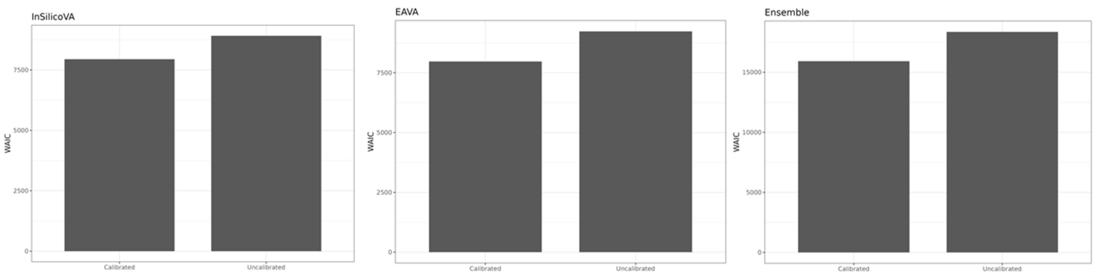

Figure S1: Model comparison for the multi-cause uncalibrated and calibrated Children (1-59 months) CSMF estimates of InSilicoVA (left), EAVA (center), and Ensemble (right) using WAIC.

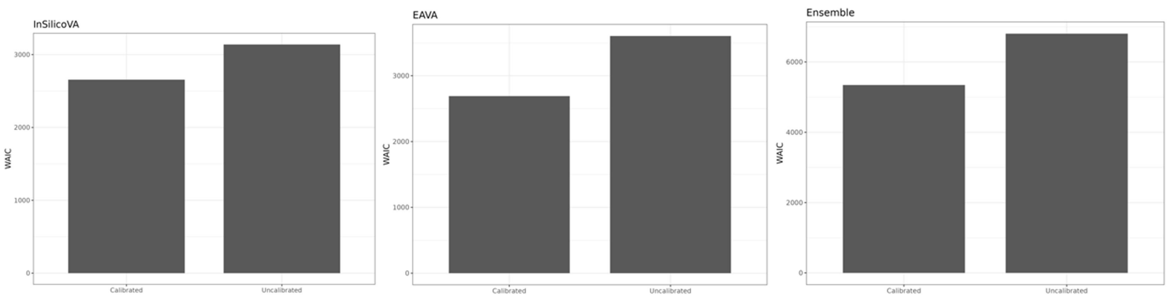

Figure S2: Model comparison for the multi-cause uncalibrated and calibrated Neonate CSMF estimates of InSilicoVA (left), EAVA (center), and Ensemble (right) using WAIC.

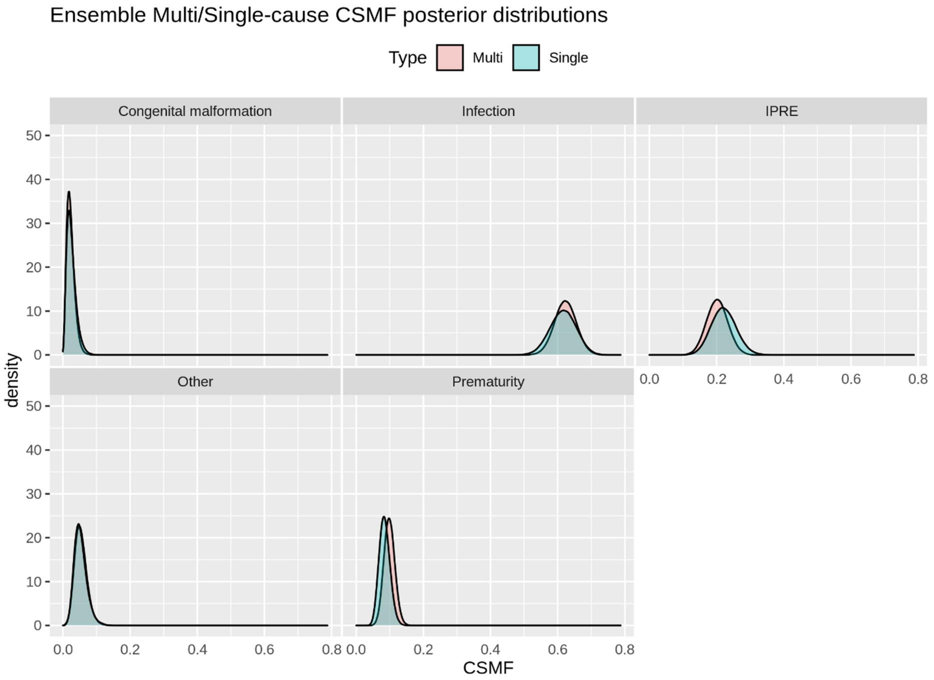

Figure S3: Comparison of the posterior densities of single- and multi-cause ensemble calibrated CSMF for neonates.

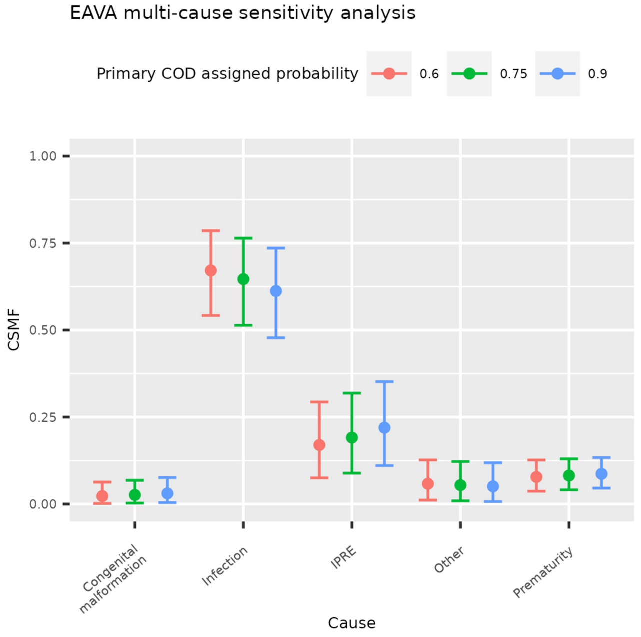

Figure S4: Sensitivity of neonate EAVA point estimates and 95% confidence intervals to adjustment of the assigned probability (weight) to the primary COD as identified by EAVA. (Main results use 0.75.)
